# Signal peptidase 21 suppresses cell proliferation, migration, and invasion via the PTEN-PI3K/Akt signaling pathway in lung adenocarcinoma

**DOI:** 10.7717/peerj.14206

**Published:** 2022-10-17

**Authors:** Na Zhang, Shiguang Cao, Ruiying Sun, Yibei Wang, Luna Liu, Wei Wang, Xia Meng

**Affiliations:** 1 Department of Pathology, The Second Affiliated Hospital, Xi’an JiaoTong University, Xi’an, Shaanxi, China; 2 Department of Nuclear Medicine, The Second Affiliated Hospital, Xi’an Jiaotong University, Xi’an, Shaanxi, China; 3 Department of Respiratory and Critical Care Medicine, The Second Affiliated Hospital, Xi’an Jiaotong University, Xi’an, Shaanxi, China; 4 Department of Physical Examination, The First Affiliated Hospital, Xi’an Jiaotong University, Xi’an, Shaanxi, China

**Keywords:** SPC21, Lung adenocarcinoma, Akt, PTEN

## Abstract

**Background:**

In a previous study, a total of 568 differentially expressed proteins including the signal peptidase SPC21 were identified from lung adenocarcinoma (LUAD) and paired normal lung tissues. In this study, the role of SPC21 in LUAD progression was investigated.

**Methods:**

The relationships and protein-protein interaction network of proteins differentially expressed between paired LUAD samples and adjacent normal tissues samples were identified via the *String* and *Pajek* software, respectively. The expression levels of the hub protein SPC21 were analyzed in 84 LUAD-normal paired tissues via immunohistochemistry. The prognostic value of SPC21 mRNA was investigated in 478 LUAD patients from TCGA and GTEx datasets. siRNAs were used in A549 and NCI-H1299 cells to knockdown SPC21. The SPC21 biological function was evaluated using the CCK-8, EdU, plate colony formation, transwell, wound healing, and adhesion assays.

**Results:**

Patients with lower SPC21 mRNA levels tended to have worse prognosis (overall survival) than those with higher mRNA levels. SPC21 expression was significantly downregulated in LUAD tumor tissues compared with that in paired normal tissues (*P* < 0.001). Functionally, SPC21 knockdown promoted cell growth, migration, and invasion. Further analyses showed that SPC21 inactivated Akt signaling, and the Akt inhibitor MK-2206 blocked the tumor-promoting effects of SPC21 knockdown.

**Conclusions:**

SPC21 plays a tumor suppressor role in LUAD cells by targeting the PTEN-PI3K/Akt axis and might be used as a prognostic indicator and therapeutic target in LUAD patients.

## Introduction

Lung cancer is still the leading cause of cancer-related deaths worldwide, with the highest morbidity and mortality rates, resulting in serious universal health matters and considerable societal burdens (Sung et al., 2021). Lung adenocarcinoma (LUAD) is the most prevalently diagnosed pathological subtype of lung cancer. The insensitivity and development of resistance to existing therapies render the discovery of new biomarkers urgent. In a previous study, we used manual microdissection to isolate the cancer target cells from LUAD tissues and matched normal tissues. A total of 568 differentially expressed proteins in the membrane structures were identified using isobaric tags for relative and absolute quantification combined with liquid chromatography-tandem mass spectrometry (Zhang et al., 2015). The possible interactions of these proteins were analyzed using bioinformatics, and several hub proteins were identified. Among these, we have already verified the expression and function of DHX9 (Cao et al., 2017; Yan et al., 2019), ADFP (Meng et al., 2021; Zhang et al., 2014), and GALNT2 (Wang et al., 2021) in LUAD *in vitro* and *in vivo* models, patient databases and tissue samples. In the present study, we analyzed the role of SPC21 in LUAD progression and evaluated its value as a prognostic indicator and therapeutic target.

The signal peptides, anchored on the N-terminus of membrane or secreted proteins guide the newly synthesized proteins to the endoplasmic reticulum. Subsequently, the signal peptides are degraded by signal peptidases (SPs), and the proteins continue to transfer to their final functional cellular allocations. If the SP is missing, the protein carrying the signal peptide cannot be correctly positioned in its functional area (Paetzel et al., 2002). SPs form a signal peptidase complex (SPC), which hydrolyze signal peptides in the endoplasmic reticulum of the eukaryotic cells. The SPC contains five subunits: SPC12, SPC18, SPC21, SPC22/23, and SPC25, and among these, SPC21 is the main catalytic site (Dalbey et al., 1997; Tjalsma et al., 2004).

Currently, the role of SPC21 in tumors have not been extensively investigated. SPC21 has been identified as a metastasis-specific gene using cDNA microarray and RT-PCR in an intrapancreatic transplantation Syrian golden hamster model, where it was downregulated and shown to be involved in cancer invasion and metastasis (Tan et al., 2010). Chai et al. (2019) compared the RNA expression of 10 parathyroid adenoma tissues with five normal parathyroid gland tissues using transcriptome analysis, and identified a densely connected submodule that included SPC21 by network analysis. These results suggest that SPC21 may play a role in the development of tumors, but its biological functions for tumor progression have not been explored yet.

In the present study, we evaluated the expression of SPC21 in LUAD tissues and paired normal tissues. In addition, we suppressed SPC21 expression in A549 and NCI-H1299 cell lines using siRNAs and evaluated its biological functions and possible mechanisms for tumor progression.

## Materials & Methods

### Analysis in silico

The data for the comparative membrane proteomic analysis between LUAD and normal tissues were obtained from our previous study (Zhang et al., 2015). The *String* software (http://string.embl.de/) was used to predict the relationship among differentially expressed proteins. The constructed protein-protein-interaction networks were visualized using the *Pajek* software (OmicX, Rouen, France) for network data integration and visualization, and the degree of interaction of each protein was calculated. Datasets from The Cancer Genome Atlas (TCGA) and Genotype-Tissue Expression (GTEx) (Tang et al., 2017) were analyzed for prognosis by the website tool *GEPIA* (http://gepia.cancer-pku.cn/).

### Tissue samples

A total of 84 tumor tissues and 84 paired adjacent non-tumor tissues from 84 LUAD patients, operated between 2018 and 2021, were used in this study. All samples were fixed in formalin and embedded in paraffin. The Institutional Review Board of the Second Affiliated Hospital of Xi’an Jiaotong University approved this study (2021084) and patients or legal guardians of patients wrote informed consents.

### Immunohistochemistry (IHC)

Sections were deparaffinized and rehydrated using xylene and a gradient of decreasing concentrations of ethanol, placed in citrate buffer and boiled for antigen retrieval for 10 min. The samples were then incubated with 3% H_2_O_2_ for 10 min, goat serum for 30 min, and a rabbit anti SPC21 antibody (1:500; Novusbio, Hubei, China) at 4 °C overnight. After rinsing with TBS buffer three times for 5 min, the sections were incubated with a goat anti rabbit biotinylated secondary antibody at room temperature for 1 h. After being washed with TBST three times for 15 min, the sections were incubated by streptomycin-HRP for 30 min. Antibody binding was visualized using DAB. Next, the sections were counterstained with hematoxylin, and washed with running water. After that, sections were dehydrated by a gradient of increased concentrations of ethanol and xylene. Finally, sections were sealed with neutral balsam and coverslipped. Two pathologists scored the samples separately and provided immunoreactivity scores ranging from 0 to 12. The immunoreactivity score was calculated as the product of the positive cell proportion score (0 means no positive cells, 1 means 1%–25%, 2 means 26%–50%, 3 means 51%–75% and 4 means 76%–100%) and the staining intensity score (0 means no color reaction, 1 means mild, 2 means moderate, and 3 means intense). Samples were divided into low (<8) or high (≥8) grades according to the final scores.

### Cell culture

Two human LUAD cell lines, A549 and NCI-H1299, were obtained from the National Collection of Authenticated Cell Cultures (Shanghai, China). Cells were cultured in RPMI-1640 medium (Gibco, Waltham, MA, USA) with 10% FBS (BI, Tel Aviv, Israel) at 37 °C and 5% CO_2_. MK-2206 (Selleck, Shanghai, China), inhibiting the phosphorylation process against Akt1, Akt2 and Akt3, was diluted with DMSO (Sigma, Waltham, MA, USA) to 20 mM. Next, the solution was diluted to 1 mM with RPMI-1640 and stored at −20 °C. Cells were treated with MK-2206 at 5 µM after culturing for 24 h, which was marked as the 0 h in MK-2206 group.

### siRNA transfection

A549 and NCI-H1299 cells were plated in 24-well plates at a density of 1 ×10^4^ cells per well in one mL of complete medium (RPMI-1640 medium with 10% fetal bovine serum). The siRNAs (GenePharma, Suzhou, China) for SPC21 (siR-1 sense: 5′-UCUUCUGCACUCAUGAUAUTT-3′, siR-1 antisense: 5′-AUAUCAUGAGUGCAGAAGATT-3′; siR-2 sense: 5′-GGGUGCAUAUGUGUUACUATT-3′, siR-2 antisense: 5′-UAGUAACACAUAUGCACCCTT-3′; siR-3 sense: 5′-CCAAUAGUUCACAGAGUAATT-3′, siR-3 antisense: 5′-UUACUCUGUGAACUAUUGGTT-3′), and control siRNA labeled with fluorescein were transfected using the X-tremeGENE siRNA Transfection Reagent (Roche, Basel, Switzerland) into cells following overnight incubation. The efficiency of transfection was evaluated by measuring fluorescence (≥ 90% of total cells).

### RT-qPCR

An RNA Extraction Kit (Takara, Kuratsu, Japan) was used to extract whole cellular RNAs, which were reverse transcribed into cDNAs by PrimeScript™ RT Master Mix Kit (Takara, Japan). The corresponding primers were designed and synthesized (Sangon Biotech, China). Quantification of mRNA was performed using TB Green^®^ Premix Ex Taq™ II Kit (Takara, Japan). The relative mRNA levels of SPC21 were normalized by GAPDH with 2^−ΔΔCt^ method. The primers sequences were as follows: SPC21 (forward 5′- AGGCCAGAACTGGCTGGAA-3′, reverse 5′- TCTGGTCCCAGGAACTGCTT-3′); GAPDH (forward 5′-GTCTCCTCTGACTTCAACAGCG-3′, reverse 5′-ACCACCCTGTTGCTGTAGCCAA-3′).

### Western blotting

All cellular proteins were extracted using lysis buffer (Beyotime, China) with phosphatase inhibitor (Roche, Basel, Switzerland). The proteins were loaded in SDS-PAGE to be separated and transferred to PVDF membranes (Millipore, Burlington, MA, USA). The PVDF membranes were blocked with 10% skimmed milk in TBST and incubated with the primary antibodies specific for a rabbit anti SPC21 antibody at 1:500 (Novusbio Biologicals, Hubei, China), a rabbit anti PTEN antibody at 1:800 (Abcam, Waltham, MA, USA), a rabbit anti p-Akt antibody at 1:1000 (CST, USA), a rabbit anti Akt antibody at 1:1000 (CST, USA), and a rabbit anti *β*-actin antibody at 1:2000 (Abcam). After being washed with TBST three times for 15 min, the membranes were incubated with secondary antibodies anti-rabbit or anti-mouse IgG biotinylated at 1:10000 (Solarbio, Beijing, China) for 2 h at room temperature. Protein bands were visualized by the ECL Chemiluminescent Kit (Millipore) and a ChemiDoc Touch imaging system (Bio-Rad, Hercules, CA, USA).

### Cell proliferation assay

In brief, both cell lines were plated in 96-well plates at a density of 6 ×10^3^ cells in 100 µL of complete medium per well. At 24, 48, 72, or 96 h, cells were then incubated with 10 µL of CCK-8 reagent (7 Sea Biotech, Shanghai, China) per well and cultured at 37 °C for 1 h and the absorbance was measured at a wavelength of 450 nm by an absorbance microplate reader (Thermo, USA).

### 5-Ethynyl-2′ -deoxyuridine (EdU) assay

Both cell lines were plated in 96-well plates at a density of 3 × 10^3^ cells per well. After starvation in RPMI-1640 medium for 24 h, cells were back to complete medium and cultured with EdU according to the instructions of EdU kit (Ribobio, Guangzhao, China) for 2 h. Cells were then stained with Apollo and DNA staining solution, according to the manufacturer’s instructions. Finally, the cells were photographed by fluorescence microscope (Leica, Japan). Cell nucleuses were stained to blue light by hoechst 33342, representing cell DNAs. Replicated DNAs were stained to red light by Apollo solution, representing proliferating cells.

### Plate colony formation

Both cell lines were seeded in six-well plates at a density of 200 cells per well and cultured for 7–10 days. Cells were then fixed in 4% paraformaldehyde (Honeywell, USA), and then stained with 0.1% crystal violet (Yantuo, China). After being washed with PBS three times, colony numbers that contained at least 50 cells was counted.

### Transwell migration and invasion assays

Briefly, both cell lines were plated in 24-well transwell inserts (Millipore, USA) coated with or without Matrigel (Corning, NY, USA) at a density of 5 × 10^5^ cells or 1.5 × 10^5^ cells for invasion assay or migration assay. 200 µL of RPMI-1640 medium was added to each insert. Complete medium (800 µL) was placed in each lower well for attraction. Then cells were incubated for 24 h (NCI-H1299 cells) or 48 h (A549 cells). Subsequently, cells migrating or invading through membranes were counted using a microscope (Nikon, Tokyo, Japan).

### Wound healing assay

Both cell lines were plated in six-well plates at a density of 1 ×10^5^ cells and cultured until reaching 100% confluence. Next, a scratch was made in each well using a 100 µL tip, and the scratched area was drawn on the plate surface with a black marker. Cells were then cultured in RPMI-1640 medium for 24 h. Finally, the scratched area was visualized under a light microscope (Nikon, Tokyo, Japan) at 0 h and 24 h following the black labeling drew on the plate.

### Statistical analysis

The difference between two groups or among multiple groups were performed to access by the Student’s *t*-test, one-way analysis of variance (ANOVA), repeated-measures ANOVA, chi-square test, Fisher’s exact test, McNemar’s test, or logistic regression, as appropriate. All cellular experiments were conducted at least in triplicate. Data were presented as a mean ± SD. All statistical tests were two-sided, and values of *P* < 0.05 were considered significantly different.

## Results

### SPC21 mRNA expression is upregulated in LUAD and related with good prognosis

**Figure 1:**
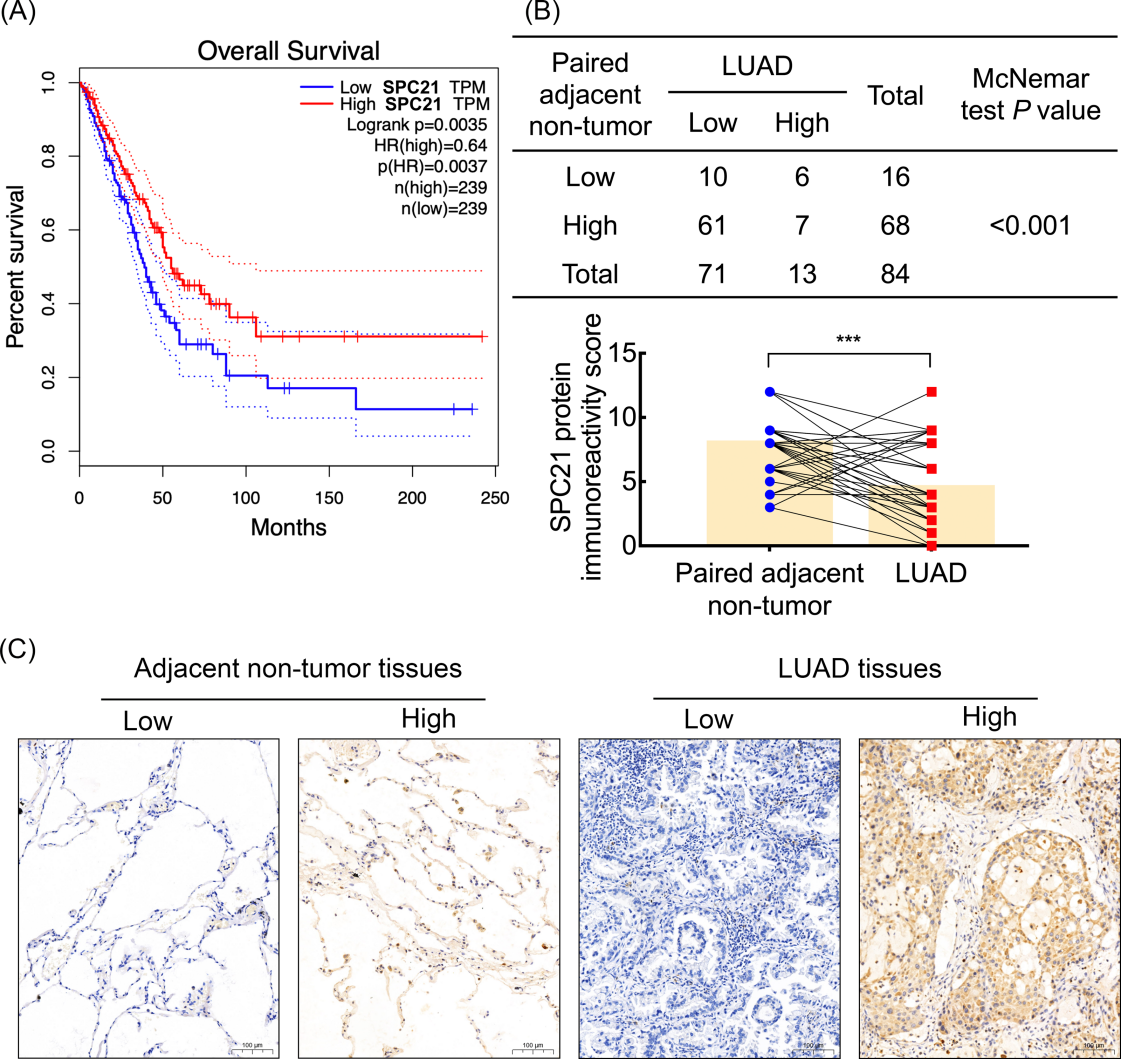
SPC21 expression analysis in lung adenocarcinoma (LUAD). (A) The high SPC21 mRNA expression group has longer overall survival than the low SPC21 mRNA expression group, as determined using the RNA-sequencing data obtained from The Cancer Genome Atlas (TCGA) and Genotype-Tissue Expression (GTEx) datasets. *P* = 0.0037. (B) Expression of SPC21 protein in tumor and paired adjacent non-tumor samples. McNemar’s test, *N* = 84, *P* < 0.001. (C) Representative images of SPC21 immunohistochemistry and counterstaining with hematoxylin in LUAD and paired adjacent non-tumor tissues showing low and high expression of SPC21. Scale bars: 100 µm.

Based on our previous comparative membrane proteomics data (Zhang et al., 2015), we analyzed the possible interactions among the differentially expressed proteins using *String* software (Fig. S1A), which showed the protein network and the connectivity degree of each protein (Fig. S1B). In line with the results of the proteomic analysis, we screened out the hub protein SPC21 and investigated the mRNA levels of *SPC21* in TCGA and GTEx datasets. Kaplan–Meier plots showed that LUAD patients with low *SPC21* expression had shorter overall survival (OS) than those with high SPC21 expression (Fig. 1A, *P* = 0.0037). Tumor tissues from 84 LUAD patients and paired adjacent non-tumor tissues were stained by IHC. The results showed lower SPC21 protein expression in LUAD than that in control tissues (Figs. 1B–1C). The analysis of the relationship between SPC21 expression in tumor tissues and the clinical paraments indicated that SPC21 was negatively associated with age and American Joint Committee on Cancer (AJCC) stage (Table 1). Furthermore, univariate logistic analysis of several variables revealed significantly low odds of high SPC21 expression in tumor tissues from elderly patients (≥60) and high AJCC stage (III/IV) (Table 2). However, after significant features were adjusted in the multivariate model, only high AJCC stage (III/IV) (OR 0.114, 95% CI [0.021–0.601], *P* = 0.010) showed significantly low odds of high SPC21 expression (Table 2).

### SPC21 suppresses proliferation, invasion, and migration in LUAD cells

**Figure 2:**
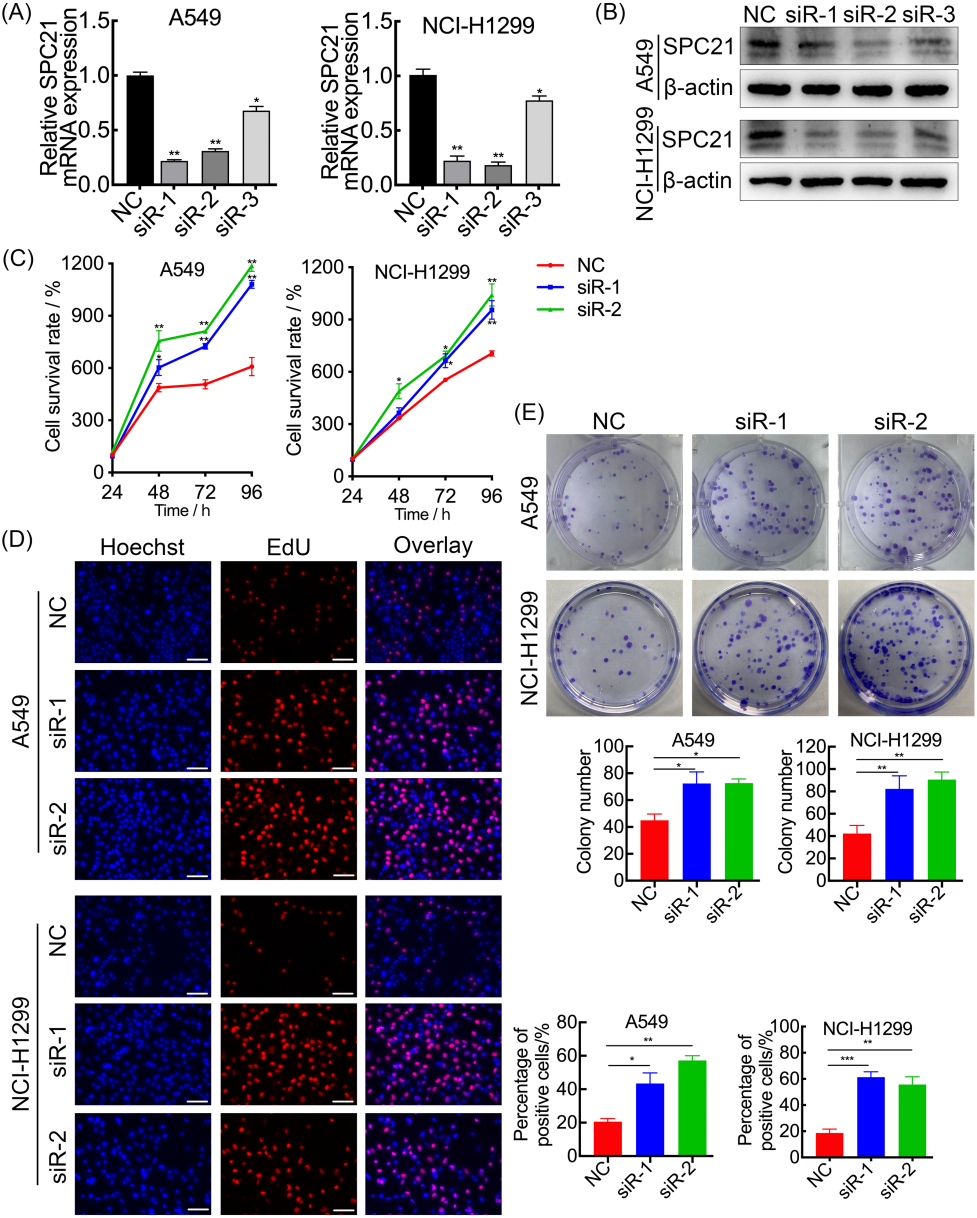
SPC21 tumor-suppressing ability on proliferation in LUAD cells. (A, B) Graphs showing mRNA (A) and protein (B) levels in A549 and NCI-H1299 cells transfected with SPC21 siRNAs measured using RT-qPCR and western-blot. (C) CCK-8 viability assay after SPC21 knockdown in LUAD cells. Graph shows the absorbance at 450 nm. (D) SPC21 knockdown substantially increased the proportion of positive cells in LUAD by EdU assay (scale bar: 50 µm). (E) SPC21 knockdown significantly increased colony numbers in LUAD cells by plate colony formation. Bars represent the SE of the mean ± SD from three independent experiments. * *P* < 0.05, ** *P* < 0.01, and *** *P* < 0.001.

**Figure 3:**
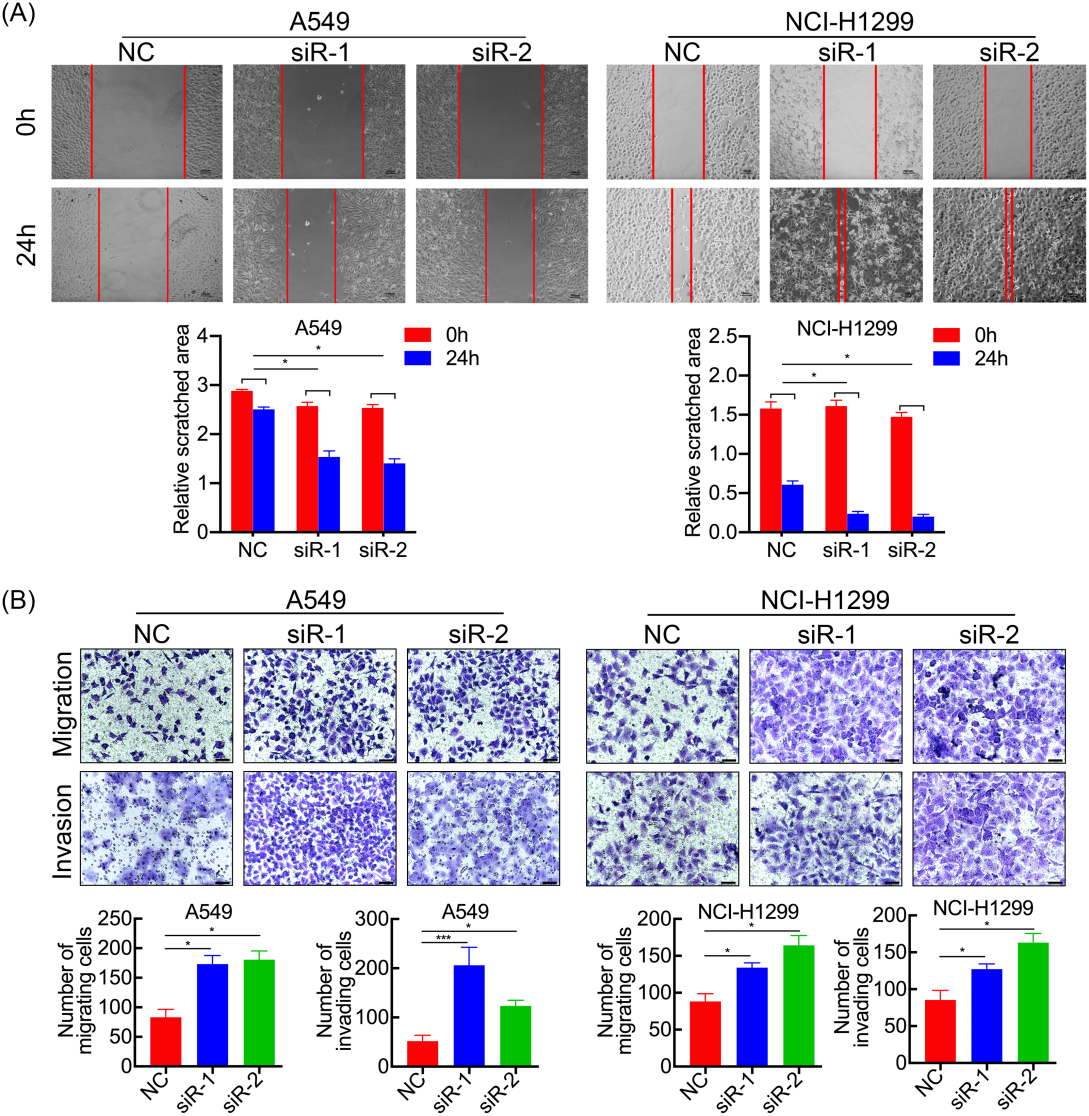
SPC21 tumor-suppressing ability on invasion and migration in LUAD cells. (A) SPC21 knockdown substantially increased the scratched areas in wound healing assays in LUAD cells (scale bar: 100 µm). (B) SPC21 knockdown significantly increased numbers of cells migrating or invading through membranes in LUAD cells by transwell assays (scale bar: 60 µm). Bars represent the SE of the mean ± SD from three independent experiments. * *P* < 0.05 and *** *P* < 0.001.

*SPC21* was knockdown using several siRNAs (siR-1, siR-2, and siR-3) in the LUAD cell lines A549 and NCI-H1299. Both siR-1 and siR-2 suppressed SPC21 expression at not only the mRNA but also protein levels in LUAD cells, whereas siR-3 did not (Figs. 2A–2B). Therefore, only siR-1 and siR-2 were used for further experiments. The CCK-8, EdU, and colony formation assays indicated that knockdown of SPC21 substantially promoted cell proliferation in A549 and NCI-H1299 (Figs. 2C–2E). In addition, the results of the wound healing and transwell assays showed that *SPC21* knockdown enhanced the migration and invasion abilities of LUAD cells (Figs. 3A–3B).

### SPC21 inhibits the PI3K/Akt pathway through PTEN in LUAD cells

**Figure 4:**
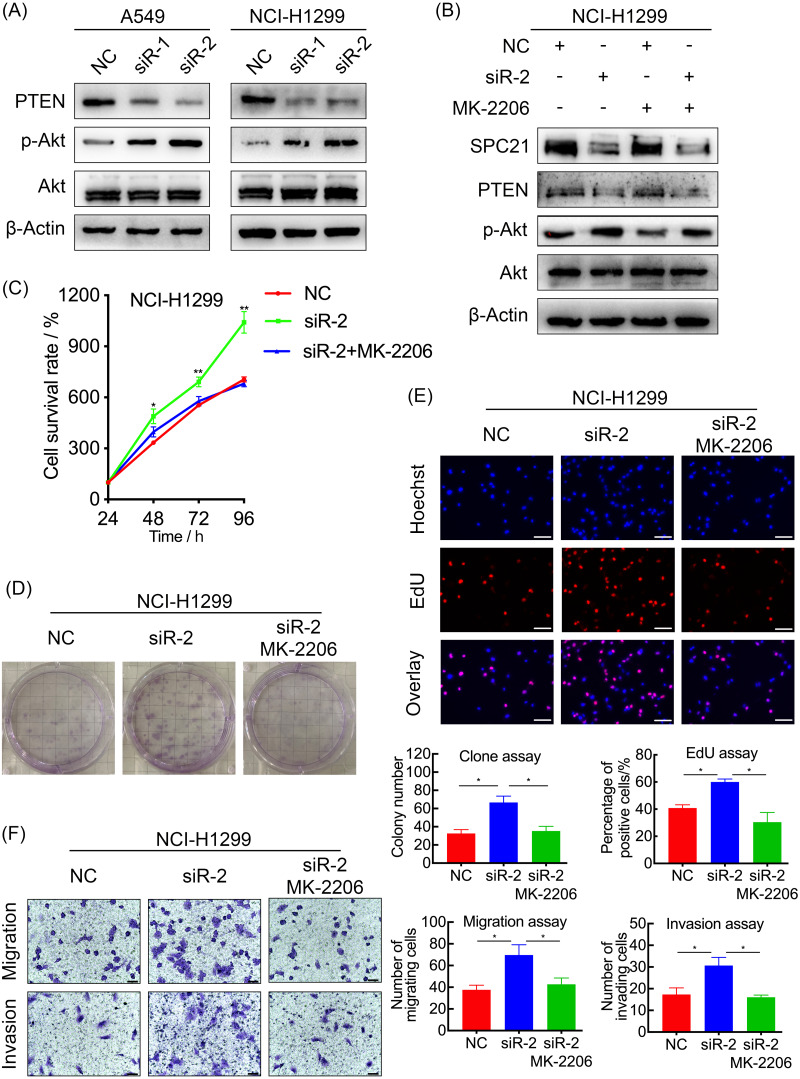
SPC21 modulates the PTEN-PI3K/Akt signaling pathway in LUAD. (A and B) Activation of the PTEN-PI3K/Akt axis in SPC21-knockdown A549 and NCI-H1299 cells (A) or MK-2206 intervened NCI-H1299 cell (B), as detected by western blotting. (C) Proliferation viability of SPC21-knockdown LUAD cells after MK-2206 treatment. (D) Number of colonies formed by SPC21-knockdown LUAD cells after MK-2206 treatment. (E) Proportion of proliferating SPC21-knockdown LUAD cells after MK-2206 treatment measured using the EdU assay (scale bar: 50 µm). (F) Migration and invasion viabilities of SPC21-knockdown LUAD cells after MK-2206 treatment measured using the transwell assays (scale bar: 60 µm). Bars represent the SE of the mean ± SD from three independent experiments. * *P* < 0.05 and ** *P* < 0.01.

In order to explore the potential mechanism, the protein levels of PTEN, and the phosphorylation level of Akt in A549 and NCI-H1299 cells with or without SPC21 were examined using western blotting. PTEN protein expression decreased in SPC21 knockdown LUAD cells, whereas p-Akt/Akt ratio increased (there is the same amount of protein but a higher phosphorylation) (Fig. 4A). As PTEN inhibited PI3K to regulate the phosphorylation of Akt, we speculated that SPC21 might regulate PTEN-PI3K/Akt pathway. To further investigate this hypothesis, NCI-H1299 cells were treated with MK-2206, an inhibitor of Akt phosphorylation. Western blotting analysis showed that MK-2206 reduced p-Akt levels but exerted no significant effect on SPC21 and PTEN protein levels. Interestingly, MK-2206 treatment rescued the increase phosphorylation in Akt induced by SPC21 knockdown (Fig. 4B) in NCI-H1299 cells and diminished the proliferation, migration, and invasion abilities back to the levels in the cells of control group (Figs. 4C–4F).

**Table 1:** Relevance between clinical characteristics and tumor tissues’ SPC21 expressions of 84 LUAD patients.

Characteristics		N	SPC21		*P*
			Low	High	
Gender	Male	47	41	6	0.439^a^
	Female	37	30	7	
Age	<60	37	27	10	0.009^a^
	≥60	47	44	3	
Smocking index	<400	56	45	11	0.203^b^
	≥400	28	26	2	
Regional lymph node invasion	Negative	33	26	7	0.242^a^
	Positive	51	45	6	
AJCC stage	I/II	23	15	8	0.010^b^
	III/IV	35	33	2	
	NA	26			

**Notes.**

a Chi-square test.

b Fisher’s exact test.

**Table 2:** Univariate and multivariate binary logistic regression for association between clinical characteristics and tumor tissues’ SPC21 expressions of 84 LUAD patients.

Characteristics		Univariate			Multivariate		
		OR	95% CI	*P*	OR	95% CI	*P*
Gender	Male	Ref					
	Female	1.594	0.486–5.229	0.441			
Age	<60	Ref			Ref		
	≥60	0.184	0.046–0.729	0.016			0.051
Smocking index	<400	Ref					
	≥400	0.315	0.065–1.531	0.152			
Lymph node invasion	Negative	Ref					
	Positive	0.495	0.150–1.632	0.248			
AJCC stage	I/II	Ref			Ref		
	III/IV	0.114	0.021–0.601	0.010	0.114		0.021–0.601	0.010

## Discussion

Here, we first found that *SPC21* significantly decreases in LUAD, and play a role as a tumor-inhibiting gene *via* the PTEN-PI3K/Akt pathway. Low level of SPC21 mRNA indicates poor prognosis. Recent studies on SPC21 in tumors have focused on gene and transcriptional levels. Chai et al. (2019) performed a differential transcriptome analysis on parathyroid adenomas and normal parathyroid gland tissues. In that study, the protein-protein interaction network analysis of differentially expressed genes revealed SPC21 as one of eight hub candidates, suggesting that it may play a crucial role in the development of parathyroid adenoma. However, parathyroid adenoma, as a benign tumor disease, provides limited evidence for the role of SPC21 in malignant tumors. Tan et al. (2010) established two pancreatic cancer cell lines with low and high potential for invasion metastasis after intrapancreatic transplantation in Syrian golden hamsters. *SPC21* expression was downregulated in pancreatic cancer cells with high potential for invasion metastasis. However, the researchers did not continue to verify the cellular and molecular mechanisms of the effect of SPC21 on the invasion and metastasis in pancreatic cancer. In this study, we analyzed TCGA and GTEx databases using the *GEPIA* website and found that high levels of *SPC21* mRNA were favorable for OS. In addition, SPC21 mRNA level was higher in LUAD than that in adjacent normal paired lung tissues. SPC21 is the main active subunit of the SPC (Dalbey et al., 1997; Tjalsma et al., 2004), and its mRNA increased in LUAD tissue, in agreement with previous observations indicating that signal peptides, the guides for the processing and transportation of secretory proteins, and the related SPs are in high demand in tumors, which might be associated with the active processes of tumor cell with an increase in the synthesis, modification, and secretion of many proteins (Gottfried, Kreutz & Mackensen, 2012). In the present study, SPC21 was higher at mRNA level in LUAD but lower at protein level, validated by mRNA database and LUAD tissue microarray, respectively. We speculate that SPC21, as an important molecule for degrading signal peptides, is transcriptionally increased in metabolically active tumor cells. This hypothesis is also supported by the fact that LUAD patients with higher mRNA levels have a better prognosis. However, SPC21 is significantly inhibited during translation, and the SPC21 protein cannot function in tumor cells due to reduced protein levels. As a result, the hydrolysis of a series of signal peptides is inhibited, and the newly synthesized proteins anchored by the signal peptides cannot be properly localized to their functional regions in cells, which may further exacerbate cellular metabolic disorders. The mechanism of this post-transcriptional regulation is unclear and requires further experimental exploration. In addition, our omics and clinical data analyses suggested that SPC21 may inhibit tumor development. Therefore, we explored the tumor suppressor effects of SPC21 and observed that *SPC21* knockdown potentiated proliferation, invasion, and migration in LUAD cells as well as the activity of the PTEN-PI3K/Akt axis. Furthermore, inhibition of Akt phosphorylation with the small-molecule inhibitor MK-2206 led to a decrease in p-Akt/Akt levels, whereas SPC21 and PTEN expression levels were not significantly altered. Interestingly, MK-2206 treatment reversed the cancer promoting effects of SPC21 knockdown. Altogether, these results suggest that SPC21 exerts a tumor suppressor effect through the PTEN-PI3K/Akt axis. However, the potential mechanism through which SPC21 regulates PTEN still needs to be further explored.

## Conclusions

In summary, SPC21 protein expression is downregulated in LUAD with a higher mRNA level. SPC21 suppresses the proliferation and metastasis of LUAD cells *via* the PTEN-PI3K/Akt signaling pathway. Our findings throw new mechanistic insights into the basic theory of LUAD progression.

##  Supplemental Information

10.7717/peerj.14206/supp-1 Supplemental Information 1 Original data for tables

10.7717/peerj.14206/supp-2 Supplemental Information 2 Original data for figures

10.7717/peerj.14206/supp-3 Supplemental Information 3 (A) The protein-protein interaction network of differential expressed proteins in LUAD analyzed by *String*. (B)The connection degree of protein-protein interaction analysis showed by *Pajek*

10.7717/peerj.14206/supp-4 Supplemental Information 4 WB original images

## Additional Information and Declarations

### Data Availability

The following information was supplied regarding data availability: The original western blotting gels images are available in the Supplemental Files.
