# Supplementary figures and images for: Signal peptidase 21 suppresses cell proliferation, migration, and invasion via the PTEN-PI3K/Akt signaling pathway in lung adenocarcinoma
Source: PeerJ. 2022 Oct 17;10:e14206. doi: 10.7717/peerj.14206 (PMC9583857; doi:10.7717/peerj.14206)

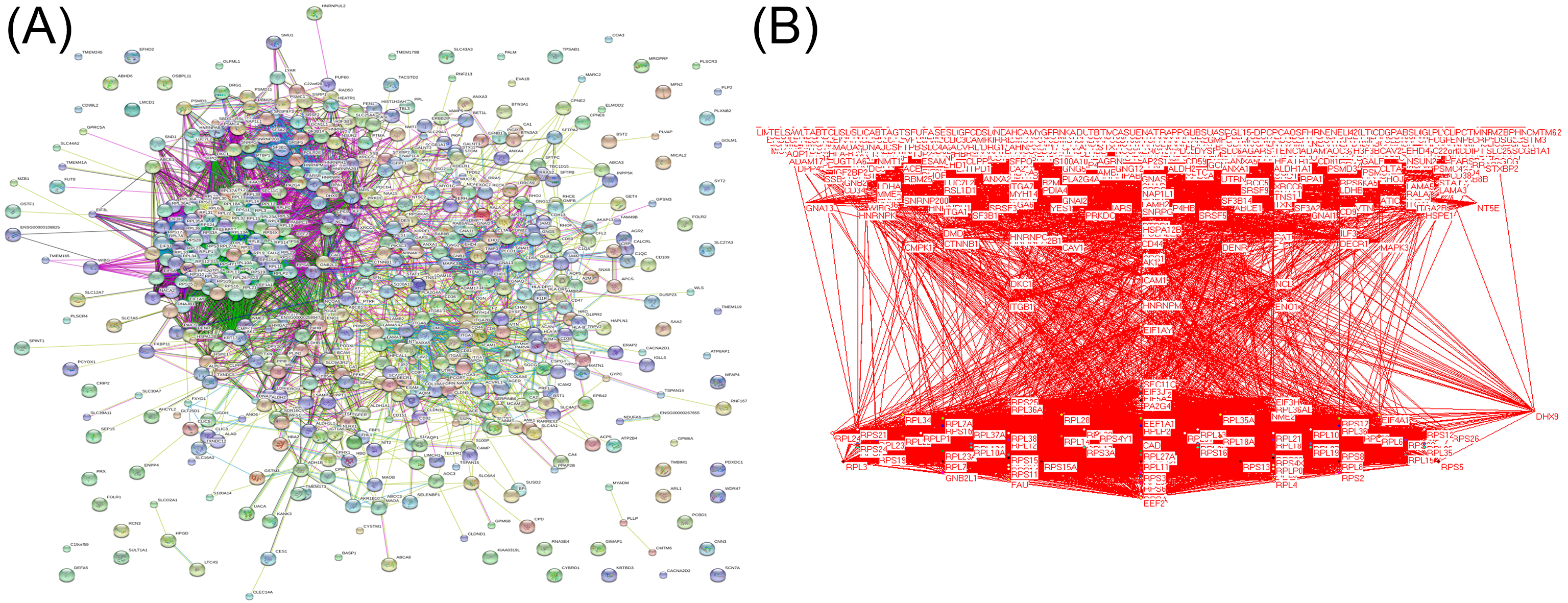

Supplement: Supplemental Information 3 [file peerj-10-14206-s003.jpg]
