# Supplementary material for: Signal peptidase 21 suppresses cell proliferation, migration, and invasion via the PTEN-PI3K/Akt signaling pathway in lung adenocarcinoma
Source: PeerJ. 2022 Oct 17;10:e14206. doi: 10.7717/peerj.14206 (PMC9583857; doi:10.7717/peerj.14206)

**WB images in figure 2B**

A549 SPC21


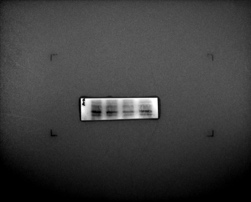


A549 𝝱-Actin


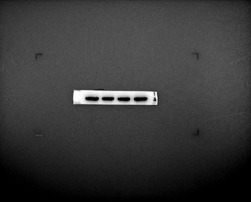


NCI-H1299 SPC21


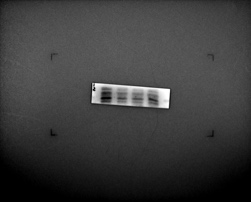


NCI-H1299 𝝱-Actin


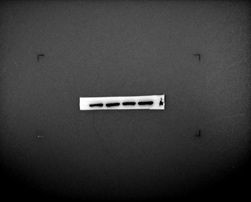


**WB images in figure 3A**

A549 PTEN


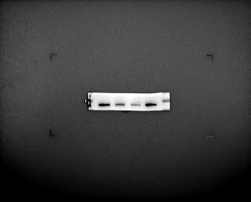


NCI-H1299 PTEN


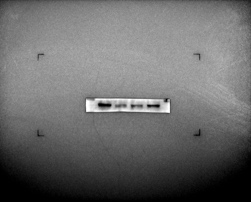


A549-p-Akt


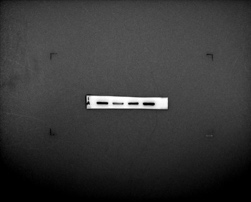


NCI-H1299 p-Akt


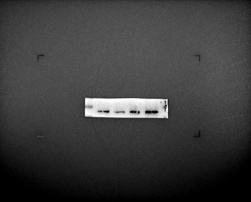


A549 Akt


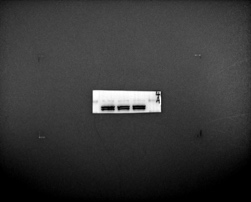


NCI-H1299 Akt


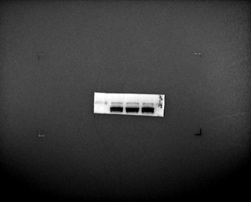


A549 𝝱-Actin


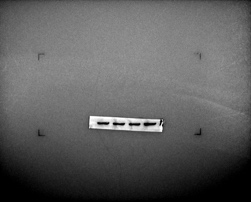


NCI-H1299 𝝱-Actin


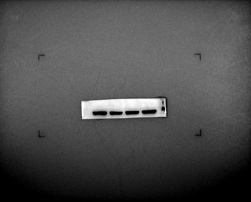


**WB images in figure 3B**

NCI-H1299+MK SPC21


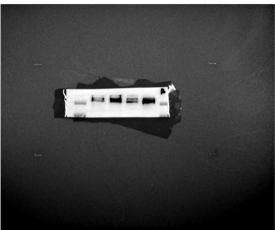


NCI-H1299+MK PTEN


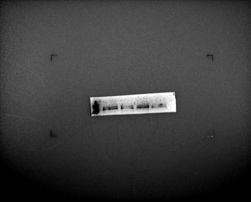


NCI-H1299+MK p-Akt


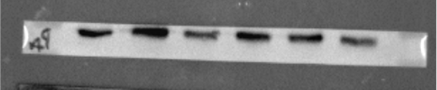


NCI-H1299+MK Akt


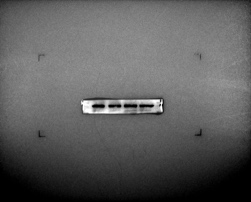


NCI-H1299+MK 𝝱-Actin


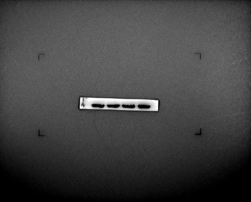

Supplement: Supplemental Information 4 [file peerj-10-14206-s004.docx]
